# Microbial community composition in alpine lake sediments from the Hengduan Mountains

**DOI:** 10.1002/mbo3.832

**Published:** 2019-03-07

**Authors:** Binqiang Liao, Xiaoxin Yan, Jiang Zhang, Ming Chen, Yanling Li, Jiafeng Huang, Ming Lei, Hailun He, Jun Wang

**Affiliations:** ^1^ School of Life Science Central South University Changsha China; ^2^ State Key Laboratory of Coal Resources and Safe Mining China University of Mining and Technology Xuzhou China; ^3^ Sanway Gene Technology Inc. Changsha China; ^4^ Key Laboratory of Plateau Lake Ecology and Environment Change, Institute of Plateau Lake Ecology and Pollution Management School of Resource Environment and Earth Science, Yunnan University Kunming China

**Keywords:** alpine lake sediments, Illumina MiSeq platform, microbial community composition, microbial diversity, the Hengduan Mountains

## Abstract

Microbial communities in sediments play an important role in alpine lake ecosystems. However, the microbial diversity and community composition of alpine lake sediments from the Hengduan Mountains remain largely unknown. Therefore, based on the Illumina MiSeq platform, high‐throughput sequencing analysis of the 16S rRNA gene was performed on 15 alpine lake sediments collected at different locations in the Hengduan Mountains. The abundance‐based coverage estimate (ACE), Chao1, and Shannon indices indicated that the microbial abundance and diversity of these sediments were high. There are some differences in the composition of microbial communities among sediments. However, in general, *Proteobacteria* accounted for the largest proportion of all sediments (22.3%–67.6%) and was the dominant phylum. Followed by *Bacteroidetes*,* Acidobacteria*,* Chloroflexi,* and *Planctomycetes*. In addition, the operational taxonomic unit (OTU) interactions network had modular structures and suggested more cooperation than competition in the microbial community. Besides, we also found that temperature has a significant contribution to the sample–environment relationship. This study revealed the diversity and composition of microbial communities in alpine lake sediments from the Hengduan Mountains, and describe the correlation between microbial community structure and different environmental variables.

## 1 INTRODUCTION

Alpine lakes are remote, hard to reach, and not impacted by human activities, so they are primitive ecosystems. Their geographic locations include many environmental conditions that are extreme for life (Catalan et al., 2006). Harsh conditions such as low temperatures, high ultraviolet radiation, and low concentrations of dissolved organic carbon and oligotrophy are characterized in these alpine environments (Čuperová, Holzer, Salka, Sommaruga, & Koblízek, 2013; Rose, Williamson, Saros, Sommaruga, & Fischer, 2009; Seufferheld, Alvarez, & Farias, 2008). These harsh conditions alter the hydrology and structure of the lake and may affect the microbial community composition and biogeochemical functions (Liu et al., 2017; Rose et al., 2009; Wasserstrom et al., 2017). Since alpine lakes have been reported to be early indicators of environmental change, the function of microorganisms in the related processes has attracted new research interest (Shafi, Kamili, Shah, Parray, & Bandh, 2017). Microorganisms are rich in genetic diversity and an essential part of aquatic ecosystems and play important roles in global biogeochemical cycles (Newton, Jones, Eiler, Mcmahon, & Bertilsson, 2011). In alpine lake ecosystems, performing these roles is supported by different microbial communities, ensuring that carbon, nitrogen, phosphorus, and sulfur are recycled back to the water column (Compte‐Port et al., 2018). Furthermore, extreme environmental variables of alpine lakes may result in species or taxon sorting that is unique to these ecosystems (Sommaruga & Casamayor, 2009). However, microbial diversity and community composition in alpine lake sediments and the associated regulatory variables need further study. Therefore, to study the diverse microbial communities of alpine lake sediments, we can understand the biogeochemical processes and ecological mechanisms that underlie ecosystem function (Liu, Yang, Yu, & Wilkinson, 2015).

The Hengduan Mountains is the general name for a series of north–south parallel mountains in Sichuan, Yunnan, and eastern Tibet in China and extending into northernmost Myanmar. There are numerous alternating ridges and deep valleys with altitudes ranging from 2,000 to 6,000 m (Oh, Wang, Wang, Liu, & Hur, 2014). These mountains are recognized as one of the 35 biodiversity hotspots in the world and the most biologically temperate region in the world (Jiménez, Long, Shevock, & Guerra, 2016). There are many lakes of different sizes in the mountain range, which are ideal places to study the microbial diversity of water bodies in the Hengduan Mountains. However, to date, there has been only sporadic knowledge of microbial communities in the area (Chen et al., 2018). Thus, profiles of the microbial diversity and community composition in alpine lake sediments of the Hengduan Mountains remain largely unknown. In addition, the correlation between microbial diversity and altitude and environmental variables in these alpine lake ecosystems remains to be studied.

In this study, we adopted integrated geochemical and molecular biological technology to analyze the microbial diversity and spatial distribution of microbial communities in remote alpine lakes. Our aims were to characterize the microbial community structure and composition in alpine lakes of the Hengduan Mountains and explore the correlation between microbial structure and different environmental variables.

## 2 MATERIALS AND METHODS

### 2.1 Sample collections and in situ measurements

**Figure 1:**
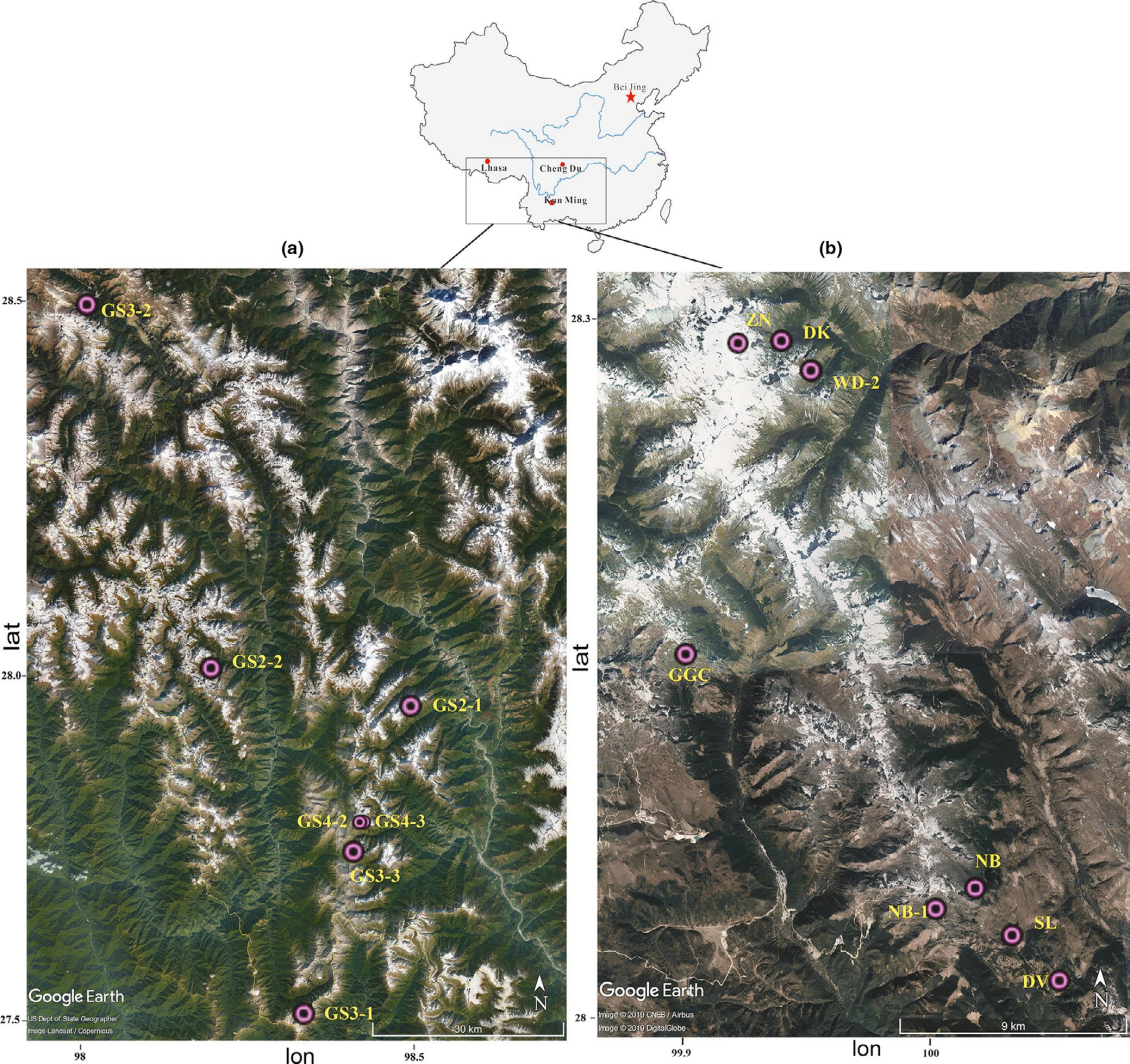
Map of the study area, showing the location of the sampling stations (a, b)

The sampling sites are located at alpine lakes in the Hengduan Mountains (27°31′40″–28°14′31.2″N and 98°12′49.2″–100°4′1.2″E), which are under the conditions of low temperature and hypoxia throughout the year. A total of 15 alpine lake sediment samples were collected using a stainless steel grab sampler at different locations in the Hengduan Mountains in 2015 (Figure 1). GS2‐1, GS2‐2, GS3‐1, GS3‐2, GS3‐3, GS4‐2, and GS4‐3 were collected in October or November; while DK, DV, GGC, NB, NB‐1, SL, WD‐2, and ZN were acquired in May or June. Samples (upper 3 cm) from each station were pooled, homogenized, collected in sterile 50 ml tubes, and immediately stored at −20°C until DNA extraction was undertaken in our laboratory. We classified the sample number based on the sampling order. At each station, depth and sediment temperature (*T*) were profiled by a SeaBird CTD (SBE37 MicroCAT, SeaBird), and altitude was determined by GPS. The pH was detected using a pH meter (Ohaus, NJ), and dissolved oxygen (DO) was measured by a DO meter (HQ30D, HACH). Sediment water content (SWC) (10 g of each sample) was determined as gravimetric weight loss after drying the sediment at 105°C until constant weight (Guo et al., 2015).

### 2.2 DNA extraction and PCR

We used next‐generation technology (16S rRNA gene‐based tag‐encoded FLX amplicon pyrosequencing, bTEFAP^®^) to characterize the microbial diversity and community composition of alpine lake sediments (Dowd, Sun, Wolcott, Domingo, & Carroll, 2008; Jacob, Hussein, Shakhatreh, & Cornelison, 2017; Swanson et al., 2011). The genomic DNA of 15 alpine lake sediments was extracted from 1 g of the sample using an E.A.N.A. Soil DNA Kit (OMEGA, Georgia, GA) as indicated in the instructions (Peng, Zi, & Wang, 2015). The V4 region of 16S rRNA gene was amplified by PCR using primers 515F (5′‐GTGCCAGCMGCCGCGGTAA‐3′) and 806R (5′‐GGACTACHVGGGTWTCTAAT‐ 3′) (Sun et al., 2015, 2017). The primer pair has been shown to generate optimal community clustering with the sequence length in the V4 region (Caporaso et al., 2011). Before sequencing on the Illumina MiSeq sequencing platform, we amplified the V4 region by adding sample‐specific 10‐base barcodes and universal sequencing tags by sample‐specific PCR protocol. Amplicon libraries were pooled with an equal volume of each barcoded product, purified using an Agencourt AMPure XP system (Beckman Coulter, CA), and the product size distribution was checked on an Agilent Bioanalyzer 2100. Purified libraries were quantified using a Qubit^®^ dsDNA HS Assay Kit (Life Technologies, CA) and then used for sequencing analysis.

### 2.3 MiSeq Illumina sequencing and data analysis

16S rRNA gene libraries were constructed using an Illumina MiSeq (San Diego, CA) platform. Filtered clean reads were analyzed using the Uparse (http://drive5.com/uparse/, version 7.1) and mothur pipelines (Version 1.35.1) (Deng, Cui, Hernández, & Dumont, 2014). All statistical analyses were carried out using the R language. The sequencing reads were classified into operational taxonomic units (OTUs; sequences with similarity ≥97% were defined as one OTU) (Peng et al., 2015). The OTU annotations were based on the Silva (Silva_119_release_aligned) database (Quast et al., 2013). Microbial α‐diversity estimates (abundance‐based coverage estimate [ACE], Chao1 and Shannon index) were calculated by the mothur program according to the OTU assignment (Star, Haverkamp, Jentoft, & Jakobsen, 2013). The relative abundance (i.e., the proportion of sequences from a phylum/class relative to the total number of sequences in the sample) was calculated. We performed a heatmap analysis using drawing tools on the BMKCloud platform (www.biocloud.net). Rarefaction curves, hierarchical clustering analysis, and principal coordinate analysis (PCoA) were performed using Past3 software (Version 3.22) (Aguilar, Acosta, Dorador, & Sommaruga, 2016). The relationship between microbial communities and individual environmental factors was analyzed by a correlation test using redundancy analysis (RDA; Canoco 5). OTU interactive network analysis was performed on the MENA website (http://ieg4.rccc.ou.edu/MENA/). The obtained sample OTU matrix distribution, according to the random matrix theory method, was used to generate the OTU interactive relationship, and further construct an ecological network (set the OTU to appear in at least nine samples). All network connection curves were in agreement with the power law model (*R* ^2^ = 0.937). Fast greedy modularity optimization was chosen to construct the module. Cytoscape software (Version 3.6.1) was used to show the network diagram, which was presented in attribute circle layout (circle by No. module) (Deng et al., 2012).

## 3 RESULTS AND DISCUSSION

### 3.1 Site descriptions and environmental variables

A total of 15 alpine lake sediment samples from different geographical locations were included in this study. The geographic location and environment variables data for each sample site are shown in Figure 1 and Table 1. These sites are located in the mountains of Yunnan and Tibet in Southwest China. All samples were collected at altitudes above 3,500 m, ZN had the highest altitude at 4,636 m, and the water depth ranged from 2.4 to 53.8 m for all sites. The temperature of most samples ranged from 7.1 to 14.6°C, except for ZN, which had the lowest temperature at 3.5°C. The sediment pH ranged from 6.03 to 9.57. Individual samples (GS2‐2, GS3‐3, and GS4‐3) had pH values below 7.0, while the other samples were neutral or alkaline. The highest DO concentration in all samples was 7.94 mg/L for GS4‐3 and the concentration in the remaining samples was approximately 7.0 mg/L. The SWC varied from 14.06% to 95.23%.

**Table 1:** Site descriptions and environmental variables

Sample ID	Longitude (E)	Latitude (N)	Depth (m)	Altitude (m)	*T* (°C)	pH	DO (mg/L)	SWC, %
GS2‐1	98°33′25.2″	27°57′32.4″	27.3	4,023	7.7	8.762	7.56	14.26
GS2‐2	98°14′34.8″	28°0′43.2″	32	4,101	7.1	6.718	7.58	28.21
GS3‐1	98°23′20.2″	27°31′40.3″	18.7	3,500	11.3	7.098	7.3	40.39
GS3‐2	98°12′49.2″	28°3′	45.6	3,709	10.3	7.34	7.418	34.43
GS3‐3	98°28′1.2″	27°45′18″	9.3	3,661	10.3	6.034	7.34	14.06
GS4‐2	98°28′33.6″	27°47′45.6″	53.8	3,848	8.8	7.162	7.58	52.94
GS4‐3	98°28′55.2″	27°47′45.6″	5	3,758	7.6	6.668	7.94	84.12
DK	99°57′18″	28°14′38.4″	8.1	4,104	10.1	8.368	7.46	26.13
DV	100°4′1.2″	28°1′1.2″	2.4	3,970	12.9	9.698	7.29	36.71
GGC	99°55′1.2″	28°7′58.8″	37.5	4,214	12.5	8.658	6.94	95.23
NB	100°1′58.8″	28°3′	32.3	4,014	11.9	8.318	7.01	22.46
NB‐1	100°1′1.2″	28°2′34.8″	3.4	4,271	7.8	8.456	7.24	81.78
SL	100°2′52.8″	28°1′58.8″	3.8	3,964	12.5	9.573	7.35	27.67
WD‐2	99°58′1.2″	28°13′58.8″	9.5	3,843	14.6	8.988	6.98	38.03
ZN	99°56′16.8″	28°14′31.2″	31	4,636	3.5	8.36	7.36	28.94

*Note*.

SWC: sediment water content; DO: dissolved oxygen.

### 3.2 Microbial richness and diversity

**Figure A1:**
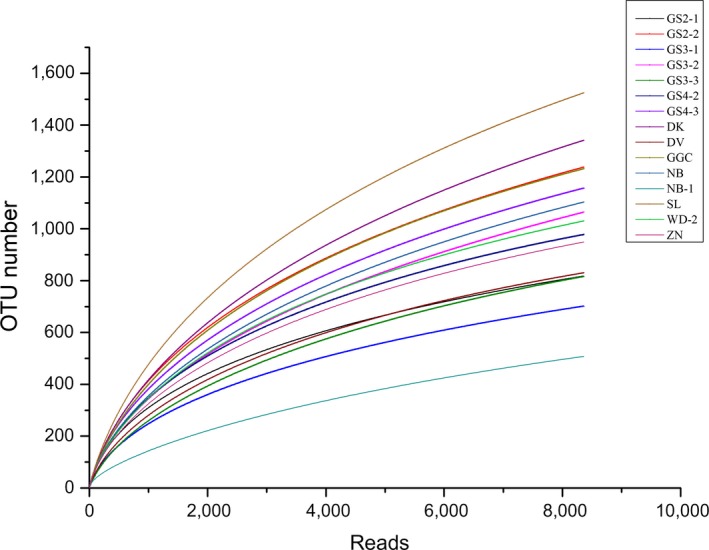
Rarefaction curves obtained from pyrosequencing data of 15 alpine lake sediments

The raw sequencing data were rigorously processed using mothur and exceeded 562,240 high‐quality partial 16S rRNA sequences that completely spanned the V4 region. After filtering, the sequence library of each sample contained 8,379–20,615 sequences (Table 2). The species abundance levels (*D* = 0.03) totaled of 15,828 OTUs in the complete data set, varying from 508 to 1,785 OTUs among sediment sample locations. The range of ACE was 1,210–2,844, while the range of Chao1 was 877–2,558, which were known as richness indices. GS4‐3 had the largest number of OTUs, correspondingly, the ACE and Chao1 index levels were also very high. In addition, NB‐1 had the fewest OTUs, and the levels of ACE and Chao1 were also low. The rarefaction curves of the OTUs indicated that diversity was completely sampled in all 15 sediments (Appendix Figure A1). The Shannon index, calculated to evaluate and compare microbial diversity among sites, was 3.62–6.54. In addition to high community richness, station GS4‐3 also had the highest microbial diversity among all sites. By comparing the two index values, we found that the diversity index was usually positively related to the richness index.

**Table 2:** Richness and diversity estimates for Illumina libraries from alpine lake sediments of the Hengduan Mountains

Sample	Number of sequences	Observed OTU	ACE	Chao1	Shannon index
GS2‐1	19,934	818	1,212	1,168	5.37
GS2‐2	19,099	1,239	2,253	1,843	5.93
GS3‐1	8,379	702	1,300	1,075	4.71
GS3‐2	19,728	962	1,811	1,486	4.61
GS3‐3	20,615	817	1,514	1,194	4.19
GS4‐2	19,314	979	1,401	1,309	5.42
GS4‐3	16,337	1,785	2,725	2,558	6.54
DK	16,206	1,343	2,602	2,088	5.92
DV	14,797	831	1,228	1,162	4.72
GGC	12,523	1,233	1,828	1,790	5.71
NB	16,768	1,104	2,065	1,691	5.53
NB‐1	18,955	508	1,210	877	3.62
SL	20,037	1,526	2,844	2,313	6.24
WD‐2	18,330	1,031	1,484	1,440	5.48
ZN	15,808	950	1,387	1,317	5.37

The results of this study indicate that the microbial abundance and diversity of the alpine lake sediments in the Hengduan Mountains are quite high. The ACE, Chao1, and Shannon indices were higher than those in previous studies in the Gossenköllesee alpine lake in Austria (Rofner et al., 2017). This suggests that the sediments from alpine lakes in the Hengduan Mountains contain more microbial diversity than we expected, and sediment microorganisms in alpine lakes have a large reservoir of genetic variability.

### 3.3 Microbial community taxonomic composition

**Figure 2:**
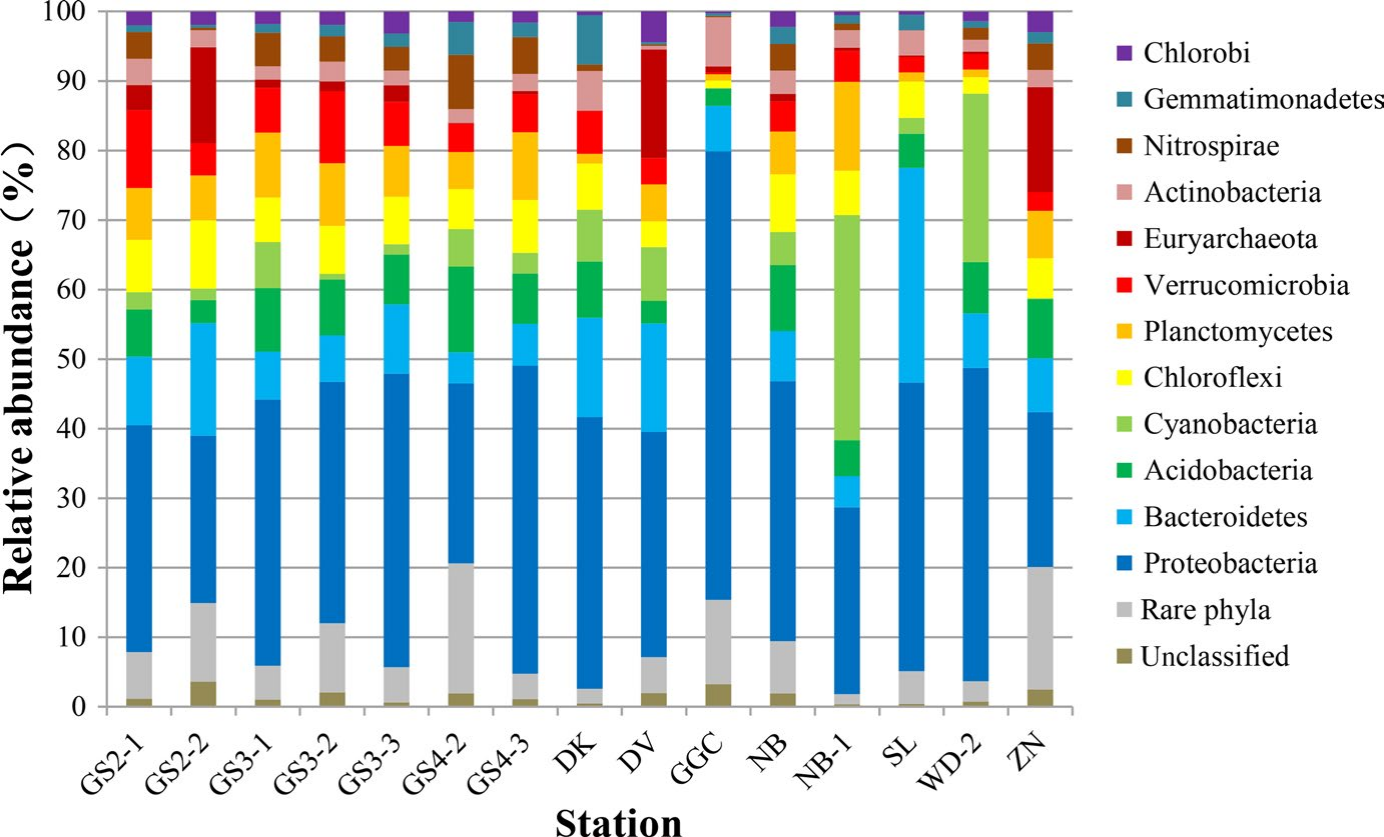
Relative abundance of phylum‐level microbial community composition for fifteen sediment samples. “Rare Phyla” include all phyla comprising <1% of the total microbial community composition

**Figure A2:**
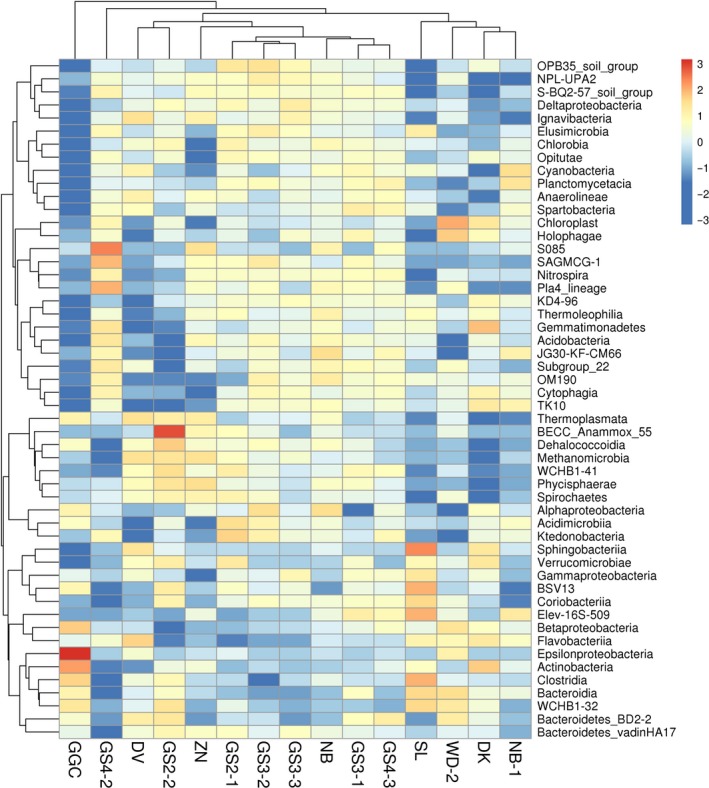
Heatmap showing the relative abundance and distribution of OTU Illumina reads. The color code indicates relative abundance, ranging from blue (low abundance) to yellow to red (high abundance). Data in Double hierarchical dendrogram were performed log10 transformation

The sequences within each Illumina MiSeq library were classified using mothur at a threshold of 45.7% to characterize the microbial community composition in each sediment sample. A total of 256,830 sequences were classified and assigned to 32 microbial phyla, 18 of which were present in each sediment sample (Figure 2; Appendix Table A1). “Rare Phyla” account for 1.45%–18.67%. OTUs that could not be assigned to a class were defined as unclassified microorganisms and were ubiquitous in all sediments but only accounted for 0.43%–3.25%. *Proteobacteria* (22.27%–64.48%) was the dominant phylum in all sediments, followed by *Bacteroidetes* (4.40%–30.85%), *Acidobacteria* (2.47%–12.32%), *Chloroflexi* (1.13%–9.82%), and *Planctomycetes* (0.87%–12.78%), which accounted for a large proportion of the phyla in most sampling stations. This result is similar to the results of microbial diversity studies in surface sediments of Qinghai Lake, Tibet (Yang, Ma, Jiang, Wu, & Dong, 2016). At the class level, *Betaproteobacteria*,* Deltaproteobacteria*,* Gammaproteobacteria*,* Acidobacteria*,* Alphaproteobacteria,* and *Sphingobacteria* were dominant, and appeared in almost all samples Appendix Figure A2).

In this study, *Proteobacteria* was the dominant phylum in all sediment samples. Many previous studies have shown that the predominance of *Proteobacteria* in various lake sediment (Aszalós et al., 2016; Dai et al., 2015; Yang et al., 2016). A variety of microorganisms from *Proteobacteria* participate in various biogeochemical processes in aquatic ecosystems (Zhang et al., 2015). Appendix Table A2 shows the 20 known proteobacterial genera detected in the 15 samples (Cheng, Zhang, Wang, Wang, & Xie, 2014). *Anaeromyxobacter* (*Deltaproteobacteria*) was found in 15 samples, and the number of sequences in most samples exceeded 50 (average 144). Microorganisms from genus *Anaeromyxobacter* are known as Fe(III) reducers (Chao, Kalinowski, Nyalwidhe, & Hansmeier, 2010), can reduce arsenate and 2‐chlorophenol (Liu, Zhang, Zhao, Zhang, & Xie, 2014), and maintain organohalide respiration (Richardson, 2013). Previous studies have shown that Alphaproteobacterial genera, such as *Bradyrhizobium*,* Hyphomicrobium*,* Sphingomonas*,* Novosphingobium*, and *Rhodanobacter* (*Gammaproteobacteria*) have the ability to degrade various environmental pollutants (Cheng et al., 2014; Liao et al., 2013). Therefore, we believe that these genera have multiple mechanisms involved in biogeochemical processes. Members of the genus *Sulfuricurvum* (*Epsilonproteobacteria*) have been considered associated with sulfur oxidation (Kodama & Watanabe, 2004). A large number of sequences classified as *Sulfuricurvum* appeared in GGC, indicating a strong sulfur oxidation process in this sediment.

In addition, we know that *Proteobacteria* and *Bacteroidetes* mainly contribute to the community structure of alpine lakes (Rofner et al., 2017). Notably, members of the *Acidobacteria* are commonly found in freshwater sediments (Newton et al., 2011). *Acidobacteria* are oligotrophic bacteria and adapt to low nutrient concentrations through strategies such as high‐affinity substrate uptake systems, low growth rates, and slow population turnover rates (Fierer, Bradford, & Jackson, 2007; Männistö, Kurhela, Tiirola, & Häggblom, 2013). Similar to several previous studies, *Bacteroidetes* exhibited large phenotypic and metabolic diversity in lake sediments (Bai et al., 2012; Liu et al. 2014; Zhang et al., 2015). Most described isolates of *Bacteroidetes* are chemoorganotrophs, known as *Cytophaga*–*Flavobacteria*, which can play an important role in converting complex molecules into simpler compounds and are also known for their phototrophic capacity (Gómez‐Consarnau et al., 2007; González et al., 2008; Kirchman, 2002; Newton et al., 2011). The archaeal diversity was much lower and dominated by *Euryarchaeota* (0.13%–45.04%). *Euryarchaeota* was mainly composed of members of the *Methanomicrobiales* and *Thermoplasmatales*. These two orders are well‐known methanogens and are involved in the central process of carbon cycling in oligotrophic alpine lake sediments (Compte‐Port et al., 2018).

Furthermore, phyla that were unique for each sampling site were observed (Appendix Table A1). For example, *Thaumarchaeota* (13.39%) was a unique dominant genus of GS4‐2, present at much higher levels than at other stations and in recent studies (Zhang et al., 2015). *Thaumarchaeota* plays an important role in ammonia oxidation, but the driving factors for its distribution in lake sediments remain unclear (Jung et al., 2014; Zhang et al., 2015). The ratio of *Cyanobacteria* at NB‐1 and WD‐2 was 32.41% and 24.22%, respectively. The unique dominant phylum in GGC was *Saccharibacteria* (7.42%). Therefore, the abundance and diversity of microorganisms in alpine lake sediments in the Hengduan Mountains are high, and there are some unique endemic species, indicating that these sediments represent promising ecosystems for the further study of biogeochemical processes.

### 3.4 Comparison of microbial community structure among sediment samples

**Figure 3:**
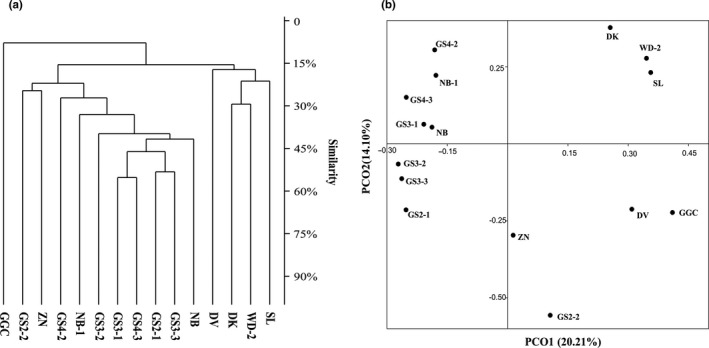
(a) Similarity unweighted pair group method with arithmetic mean dendrogram of OTU clustering analysis; (b) principal coordinate analysis based on a Bray–Curtis similarity matrix between sediment samples

An OTU matrix based hierarchical cluster using the unweighted pair group method with arithmetic mean was generated (Figure 3a). The dendrogram showed that the 15 sediment samples could be divided into five groups. The members of the first group included only GGC; the second group included GS2‐2 and ZN; the third group consisted of GS4‐2, NB‐1, GS3‐2, GS3‐1, GS4‐3, GS2‐1, GS3‐3, and NB; DV alone was included in the fourth group; and DK, WD‐2, and SL were included in the fifth group (Figure 3a). Similar results were also found in the PCoA based on a Bray–Curtis similarity matrix (Figure 3b). As shown by the cluster analysis, the sediment samples (except for GS2‐2) distributed in Figure 1a are closely grouped compared to the samples distributed in Figure 1b. This grouping means that the microbial community structure of alpine lake sediments in different locations also has spatial heterogeneity, which is consistent with previous reports (Bai et al., 2012; Chen et al., 2015; Dai et al., 2015; Song, Li, Du, Wang, & Dinget al., 2015). This spatial heterogeneity may be related to scale; spatial distance plays an important role in the formation of microbial community composition differences in the range of 10–1,000 km (Martiny, Eisen, Penn, Allison, & Horner‐Devine, 2011; Martiny et al., 2006).

### 3.5 Correlation network analysis of microbial communities

**Figure 4:**
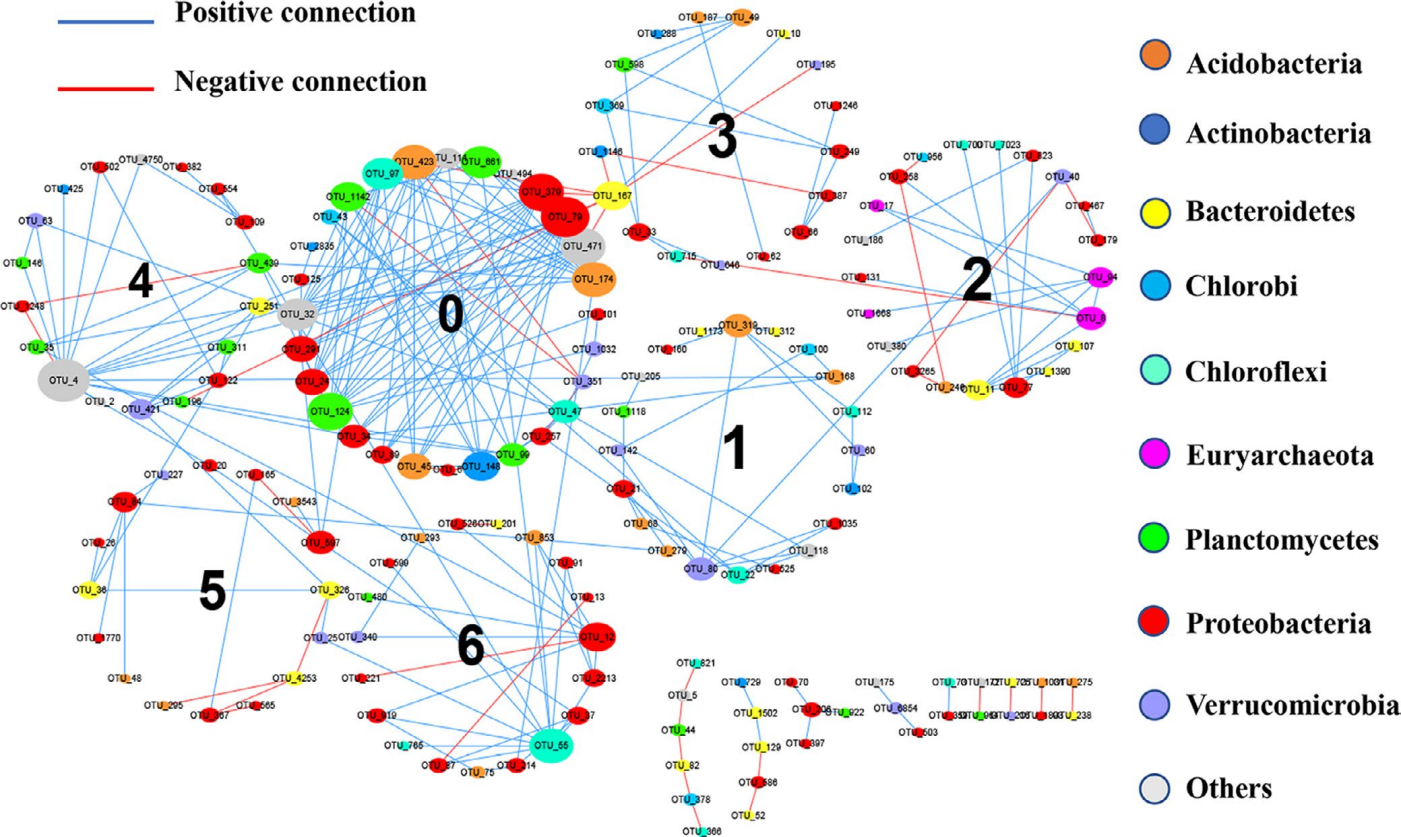
OTU interactions network according to the DNA data set of the alpine lake sediments. Each node is represented by an OTU indicating an individual species. The edge between each of the two nodes represents positive (blue) or negative (red) interactions between those two species. The colors of the nodes indicate the different major phyla and the size of each node is proportional to the number of connections (i.e., the degree). The circles consist of some nodes’ mean modules

Addressing the interactions of microbial communities in the inland water ecosystem is a longstanding challenge in microbial community ecology (Cao et al., 2018; Zhao et al., 2016). The sediment microbial network consisted of 167 nodes, and 256 edges (Figure 4; Appendix Table A3). The modularity value of the networks was 0.687(>0.50) (Appendix Table A3), and thus the networks had modular structures (Newman, 2006). Therefore, the interactions network of OTUs was divided according to the modules. Of the 256 interactions, 216 (84.4%) were positive and 40 (15.6%) were negative.

**Figure A3:**
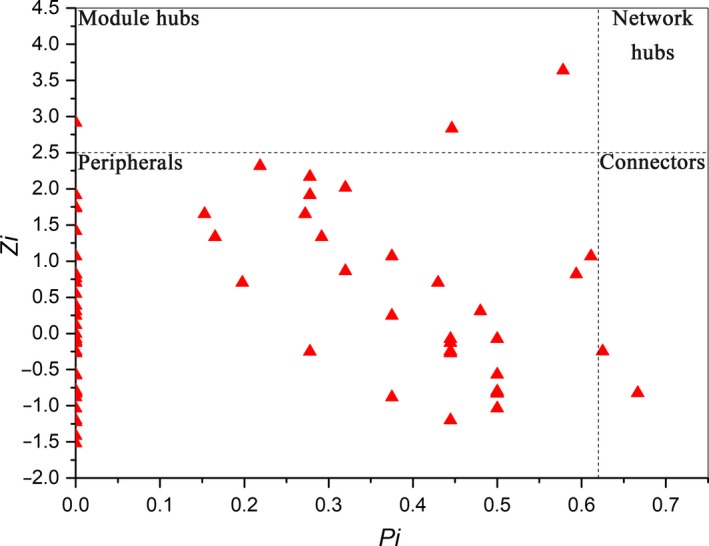
The Z–P plot showing node categories according to within‐module connectivity (Zi) and among‐module connectivity (Pi)

OTUs from the *Proteobacteria* and *Bacteroidetes* were almost universally present in all modules (Figure 4), which indicate that *Proteobacteria* and *Bacteroidetes* can accommodate different ecological environments, and this accommodation can also explain the reasons for their high abundance in all alpine lake sediments (Appendix Table A1). However, there was very little connection between *Proteobacteria* and *Bacteroidetes*, and most of the connections were negative. As previous studies have shown, species with similar ecological niches may compete when resources are scarce (Cao et al., 2018; Zhao et al., 2016). Notably, positive connections dominated the interactions in the OTUs‐based networks, suggesting more cooperation rather competition in the microbial community structure (Chow, Kim, Sachdeva, Caron, & Fuhrman, 2014; Yang et al., 2017; Zhang, Zhao, Dai, Jiao, & Herndl, 2014). In communities, positive associations may be the result of cooperation among microorganisms during long‐term coevolution, including the exchange of metabolites such as elements, nutrients, and electrons, or the coordinated decomposition of complex polymers by multiple microorganisms (Raes & Bork, 2008; Zhang et al., 2014). Due to the diverse metabolic mechanisms of *Proteobacteria*, metabolic cooperation with other microorganisms can occur. Moreover, *Proteobacteria* displayed tighter and more positive connections than the other microbial taxa, so we believe that *Proteobacteria* play a positive role in promoting the growth of the microbial community. To illustrate the topological roles of nodes a ZP‐plot was constructed. (Appendix Figure A3) (Deng et al., 2012; Olesen, Bascompte, Dupont, & Jordano, 2007). Here, most OTUs (97.0%) were peripheral, and most of their links were within their own modules. Only two nodes (OTU_168 and OTU_251) were connectors that “glue” modules together, which are important for network coherence (Olesen et al., 2007). They were derived from *Candidatus Solibacter* (*Acidobacteria*) and *Saprospiraceae* (*Bacteroidetes*). Three nodes (OTU_55, OTU_319, and OTU_4) were module hubs and important to the coherence of their own module. The three module hubs OTUs were derived from *KD4‐96* (*Chloroflexi*), *Subgroup_6* (*Acidobacteria*), and *Chloroplast* (*Cyanobacteria*). It is worth mentioning that OTU_4 (phylum‐*Cyanobacteria*) was identified as a module hub in the network of Module 4 and had the maximum number of connections (15 positive connections and one negative connection) (Figure 4), showing the importance of its microbial composition throughout the ecosystem. *Cyanobacteria* are generally considered to be oxygenic photosynthetic bacteria (Shylajanaciyar et al., 2015). OTU_4 may be highly connected with other microorganisms by providing oxygen to aerobic microorganisms, such as *Planctomycetales* (OTU_146 and OTU_99) and *Xanthomonas* (OTU_20 and OTU_99), and cooperates with other cyanobacteria to produce oxygen. This may be one reason why it was a network module hub, that is, highly connected species linked to many species within their own module (Olesen et al., 2007). Furthermore, modularity is an indicator of resistance in a system (Carpenter et al., 2012; Ding et al., 2015), and modularity is very important for the system stability of the network (Olesen et al., 2007), which might explain why microbial communities adapt to the extreme environments of high mountains.

### 3.6 The relationship between the microbial community and environmental variables

**Figure 5:**
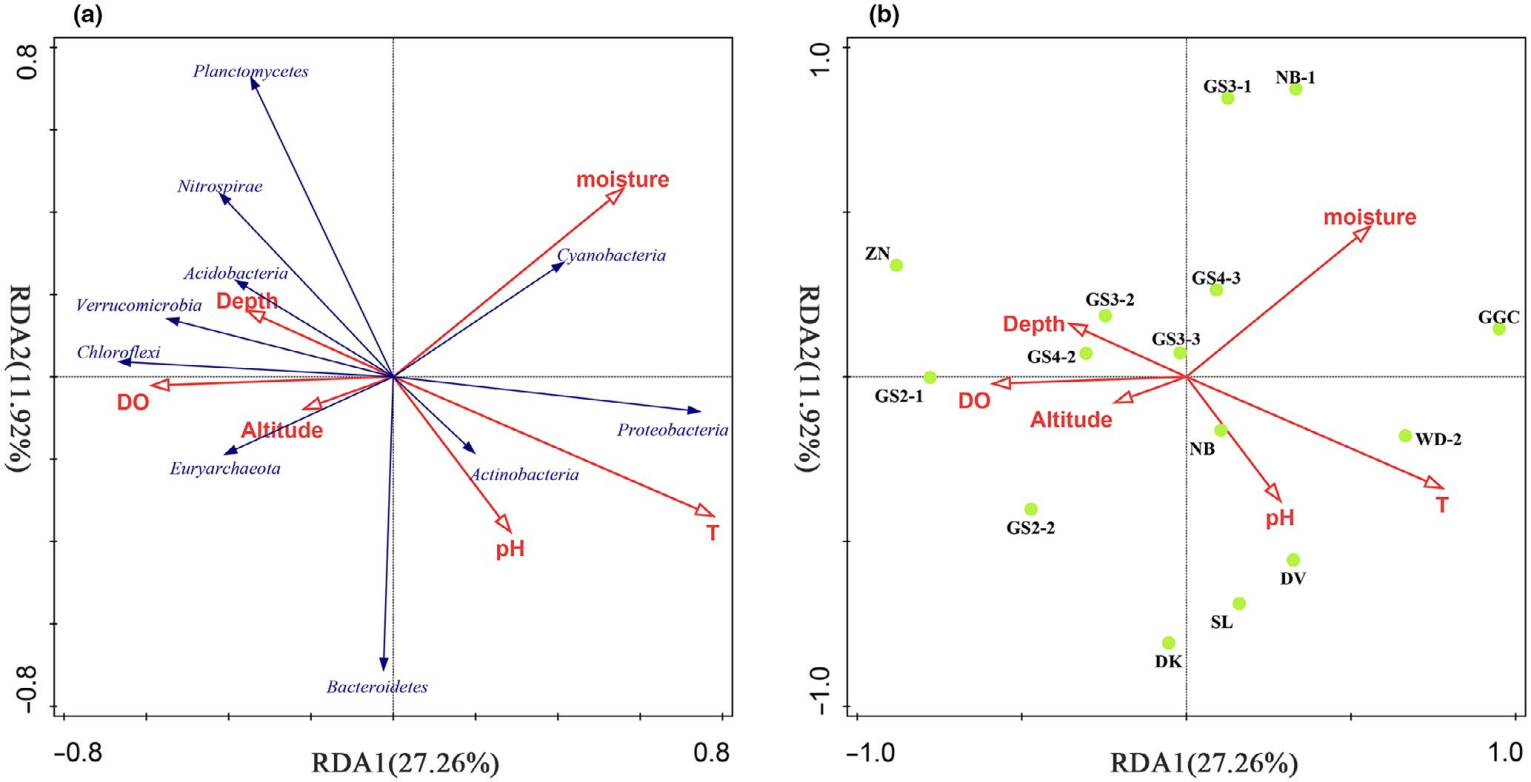
The redundancy analysis (RDA) of the relationship between microbial phyla (a), samples (b) and sediment environmental variables. Environmental variables include depth, altitude, *T*, pH, dissolved oxygen (DO), and sediment water content. Microbial phyla include: *Acidobacteria*,* Actinobacteria*,* Bacteroidetes*,* Chlorobi*,* Chloroflexi*,* Cyanobacteria*,* Euryarchaeota*,* Gemmatimonadetes*,* Nitrospirae*,* Planctomycetes*,* Proteobacteria,* and *Verrucomicrobia*

To assess the relationship between microbial communities and environmental variables, RDA analysis was performed (Figure 5a). The total interpretable variation of the microbial community composition of the first two axes RDA1 and RDA2 reached 39.18%. RDA1 was affected mainly by DO, depth, temperature, and altitude. The pH and SWC had the largest effect on RDA2. *Proteobacteria*,* Cyanobacteria,* and *Actinobacteria* were projected in the positive direction of RDA1, so these microbial phyla are positively correlated with temperature, SWC, and pH, while negatively correlated with altitude, depth, and DO. However, the correlations of the other phyla (except the *Bacteroidetes*) were reversed. In addition, *Bacteroidetes* were positively related to pH but negatively related to SWC. However, *Planctomycetes* showed the opposite correlations. RDA also indicated that only temperature contributed significantly to the sample–environment relationship (*p *<* *0.05), providing 18.4% of the RDA explanatory power (Figure 5b). Moreover, the three samples with the greatest temperature influence were GGC, WD‐2, and DV, and the least affected was ZN. Although not significant (*p *=* *0.06), SWC also provided 11.6% of the explanatory power, and GGC, NB‐1, and GS3‐1 were the three most affected samples.

**Figure 6:**
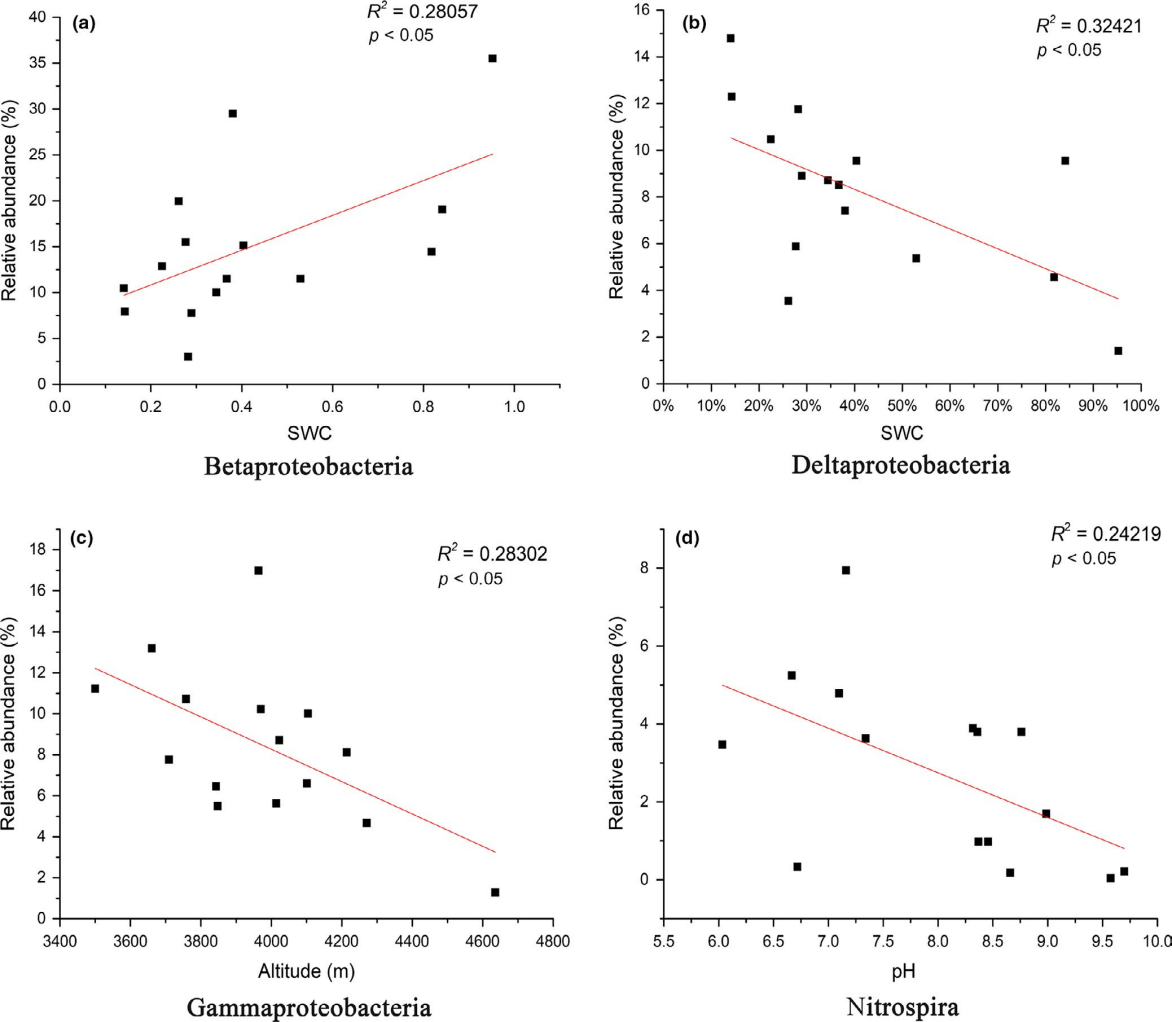
Relationships between the relative abundances of 10 dominant bacterial phyla and environmental variables (a–d). Linear or quadratic regressions were used to test the relationship between the taxa relative abundances and soil pH. Adjusted *R* ^2^ values with the associated *p*‐values are shown for each taxonomic group

To test and verify the relationship between environmental variables and microbial community composition, a correlation analysis between sediment environmental variables and the relative abundance of 10 abundant classes was performed (Xu et al., 2014) (Figure 6). The results indicated that the relative abundance of *Nitrospira* was negatively related to pH (Figure 6d). For SWC, the relative abundance of *Deltaproteobacteria* had a significant negative correlation, while SWC was positively correlated with *Betaproteobacteria* (Figure 6a,b). We also found that the relative abundance of *Gammaproteobacteria* decreased significantly with increasing altitude (Figure 6c).

The RDA results in this study suggest that temperature (*p *<* *0.05) significantly influences the microbial community composition in the alpine lake sediments (Figure 5a). Zhou, Deng, et al. (2016) described the effect of temperature on increasing microbial richness and diversity and shaping microbial community composition. High temperatures mean high metabolic rates, growth rates, ecosystem productivity, ecological interactions, and population doubling times (Brown, Gillooly, Allen, Savage, & West, 2004; Chen, Landry, Huang, & Liu, 2012; Wang, Brown, Tang, & Fang, 2009; Zhou, Deng, et al., 2016). Concurrently, other environmental factors such as water, carbon, nutrient availability, and pH can also interact with temperature to indirectly affect biodiversity and microbial community composition (Zhou, Deng, et al., 2016). Consistent with our RDA results, temperature was positively correlated with *Cyanobacteria* and *Proteobacteria*. We speculate that in higher temperature environments, due to the higher rates of metabolism and growth rates, the production and consumption of resources accelerates, so the abundance of *Cyanobacteria* and *Proteobacteria* also increases, and the chemical cycle is more borne by these two microorganisms. This speculation agrees with previous reports that temperature is a crucial environmental variable that controls the distribution of nitrogen‐fixing microbial communities, such as N_2_‐fixing *Cyanobacteria* (Stal, 2009; Zhou, Dang, & Klotz, 2016). In addition, temperature also affects the O_2_ solubility in the pore water of the sediment (Zhou, Dang, et al., 2016). In low‐temperature environments, however, anaerobic and/or facultative anaerobic microorganisms with low oxygen demand such as *Euryarchaeota*,* Chloroflexi,* and *Nitrospirae* bacteria also participate in chemical cycles. Thus, the microbial composition of alpine lake sediments is affected. Sediment SWC (Guo et al., 2015), pH (Zhao et al., 2011), DO (Yadav, Khardenavis, & Kapley, 2014), depth (Zhang et al., 2015), and geographic factors such as altitude (Chen et al., 2017) can also influence the composition of microbial communities. From the sample–environment relationship (Figure 5b) we found that the two groups that are geographically close (DK, WD‐2, and ZN; NB‐1 and SL) are distributed far apart in the figure. The environmental variables in the groups are very different, especially temperature and SWC, which suggests that local environmental variables are critical for shaping microbial community composition on a small spatial scale (Aguilar et al., 2016).

## 4 CONCLUSIONS

In this study, details of microbial diversity and community composition in 15 sediments from alpine lakes in the Hengduan Mountains were revealed. The composition of microbial communities in sediments of alpine lakes showed some specificity. In most sediment samples, *Proteobacteria*,* Planctomycetes*,* Acidobacteria*,* Bacteroidetes,* and *Chloroflexi* were abundant, while *Cyanobacteria*,* Euryarchaeota*,* Actinobacteria,* and *Nitrospirae* were only abundant in some alpine lake sediment samples. The profiles of the microbial communities in these alpine lake sediments are related to their hydrological and physicochemical properties. Temperature is one of the most critical environmental variables. Further research is necessary to clarify the relationship between environmental variables and microbial communities in alpine lake sediments.

## CONFLICT OF INTERESTS

The authors declare no conflict of interest.

## AUTHORS CONTRIBUTION

H.H.L. and W.J. conceived and designed the experiments; L.Y.L. collected the samples; L.B.Q., Y.X.X., Z.J., and C.M. performed the experiments and analyzed the data; L.B.Q. and H.H.L. wrote most of the manuscript, and all the authors assisted in writing the manuscript, discussed the results, and commented on the manuscript.

## ETHICS STATEMENT

None required.

## Data Availability

The raw sequence data have been deposited in BIGD with Genome Sequence Archive (GSA) number CRA001189 (http://bigd.big.ac.cn/gsa/s/xWfbv74D).

## APPENDIX 1

### 1.1

**Table A1:** Alpine lake sediment samples species abundance (%) at the phylum level

	GS2‐1	GS2‐2	GS3‐1	GS3‐2	GS3‐3	GS4‐2	GS4‐3	DK	DV	GGC	NB	NB‐1	SL	WD‐2	ZN
Unclassified	1.16	3.66	1.01	2.05	0.63	1.93	1.11	0.47	1.95	3.25	1.93	0.37	0.43	0.72	2.49
Proteobacteria	32.65	24.11	38.23	34.72	42.2	25.9	44.31	39.05	32.34	64.48	37.43	26.92	41.5	45.09	22.27
Bacteroidetes	9.81	16.11	6.95	6.66	9.94	4.48	5.97	14.3	15.55	6.49	7.18	4.4	30.85	7.78	7.82
Acidobacteria	6.78	3.33	9.09	8.08	7.14	12.32	7.28	8.1	3.32	2.47	9.44	5.19	4.95	7.38	8.46
Cyanobacteria	2.49	1.69	6.6	0.85	1.48	5.39	2.95	7.49	7.67	0.11	4.79	32.41	2.29	24.22	0.1
Chloroflexi	7.47	9.82	6.46	6.87	6.89	5.75	7.61	6.6	3.78	1.13	8.32	6.35	5.19	2.41	5.73
Planctomycetes	7.45	6.41	9.31	9	7.27	5.36	9.76	1.41	5.24	0.87	6.19	12.78	1.34	1.1	6.87
Verrucomicrobia	11.22	4.67	6.42	10.36	6.3	4.08	5.42	6.13	3.8	0.23	4.24	4.5	2.22	2.21	2.69
Euryarchaeota	3.6	13.77	1.17	1.38	2.43	0.13	0.48	0.05	15.65	0.9	1.11	0.44	0.18	0.39	15.04
Actinobacteria	3.84	2.43	1.96	2.86	2.11	1.91	2.46	5.72	0.53	7.04	3.39	2.46	3.59	1.69	2.54
Nitrospirae	3.8	0.33	4.77	3.62	3.43	7.88	5.28	0.97	0.21	0.18	3.85	0.98	0.04	1.69	3.83
Gemmatimonadetes	0.99	0.43	1.25	1.66	1.9	4.68	2.1	7.05	0.3	0.39	2.4	1.18	2.2	0.92	1.58
Chlorobi	1.97	1.95	1.84	1.92	3.17	1.52	1.62	0.55	4.42	0.27	2.26	0.54	0.54	1.44	3
Other	0.5	1.42	0.29	0.97	0.56	0.42	0.37	0.11	0.61	0.24	0.75	0.06	0.13	0.45	1.27
Aminicenantes	0.06	0.37	0.08	0.04	0.04	0	0.1	0	0.24	0.17	0.14	0.04	0	0.07	0.92
Armatimonadetes	0.21	0.42	0.23	0.37	0.21	0.57	0.33	0.01	0.2	0.12	0.6	0.18	0.42	0.02	0.53
Candidate_division_OP3	0.43	0.24	0.18	0.25	0.26	0.21	0.1	0	0.56	0.25	0.6	0.04	0	0.06	0.7
Elusimicrobia	0.39	0.23	0.2	0.55	0.35	0.42	0.13	0.06	0.11	0.01	0.18	0.14	0.54	0.05	0.06
Firmicutes	0.11	0.68	0.12	0.02	0.08	0.01	0.1	0.12	0.2	1.86	0.14	0.07	3.07	0.16	0.21
Latescibacteria	0.25	1.11	0.49	0.18	0.19	1.21	0.68	0.06	1.11	0.21	1.24	0.3	0.07	0.51	0.91
Lentisphaerae	0.58	1.11	0.5	0.16	0.17	0.02	0.23	0	0.27	0.02	0.17	0.01	0.01	0.13	0.2
Miscellaneous_Crenarchaeotic_Group	0.21	0.13	0.02	0.14	0.2	0	0.11	0	0.02	0.49	1.32	0.02	0	0.02	6.52
Miscellaneous_Euryarchaeotic_Group	0.02	0.23	0	0.01	0	0	0	0	0.01	0.05	0.05	0	0	0	0.57
Omnitrophica	0.26	0.18	0.14	0.51	0.35	0.19	0.08	0	0.1	0.02	0.23	0	0	0.16	0.23
Parcubacteria	0.61	1.27	0.24	0.82	0.49	0.41	0.23	0.25	0.16	0.05	0.29	0	0.11	0.38	0.32
Saccharibacteria	0.19	0.17	0.11	0.41	0.13	0.37	0.14	1.44	0.18	7.42	0.3	0.32	0.11	0.01	0.02
Spirochaetae	0.81	0.79	0.21	0.36	0.08	0.16	0.17	0	0.42	0.08	0.23	0.05	0	0.32	0.76
TA06	0.04	0.64	0.01	0.02	0.01	0.04	0.02	0	0.18	0.06	0.11	0	0	0.13	0.61
TM6	0.1	0.18	0.04	0.51	0.1	0.05	0.05	0.04	0.05	0.04	0.21	0.01	0.05	0	0.16
Thaumarchaeota	0.67	0.24	0.82	2.41	0.33	13.39	0.19	0.01	0.12	0.04	0.23	0	0	0.07	1.1
WCHB1‐60	0.25	0.2	0.42	0.84	1.43	0.02	0.18	0	0.02	0.01	0.11	0.2	0.19	0.08	0.06
Woesearchaeota_(DHVEG‐6)	1.06	1.67	0.82	1.38	0.11	1.18	0.44	0	0.69	1.03	0.58	0.01	0	0.33	2.45

**Table A2:** Distribution of the sequences affiliated with the identified proteobacterial genera in sediment samples

	GS2‐1	GS2‐2	GS3‐1	GS3‐2	GS3‐3	GS4‐2	GS4‐3	DK	DV	GGC	NB	NB‐1	SL	WD‐2	ZN	Total	Nmber
Alphaproteobacteria																	
Hyphomicrobium	1	2	0	3	1	1	0	12	0	2	5	1	2	4	2	36	12
Bradyrhizobium	0	0	0	1	0	0	1	0	3	2	0	0	0	4	1	12	6
Sphingomonas	0	1	0	1	1	0	0	90	1	1	3	1	6	2	1	108	11
Roseomonas	3	1	1	1	0	0	2	0	5	1	0	6	0	0	5	25	9
Rhodoplanes	3	0	0	1	1	0	0	2	0	0	0	0	0	1	0	8	5
Phenylobacterium	0	0	0	0	0	0	0	3	6	0	0	0	3	1	12	25	5
Novosphingobium	0	11	1	4	5	0	9	12	2	0	1	3	5	0	6	59	11
Defluviicoccus	2	1	0	8	0	0	0	8	46	0	1	0	2	2	2	72	9
Betaproteobacteria																	
Massilia	0	1	1	20	12	4	52	2	32	3	51	26	44	7	0	255	13
Vogesella	0	0	0	0	1	1	9	0	0	0	0	0	0	0	0	11	4
Gammaproteobacteria																	
Rhodanobacter	0	0	0	0	0	23	1	0	0	654	7	0	7	111	1	804	7
Methylocaldum	0	0	294	0	1	1	14	0	323	0	6	42	1	2	0	684	10
Acinetobacter	0	2	0	0	0	1	0	0	2	0	0	0	0	0	6	11	4
Steroidobacter	0	0	0	0	0	0	1	0	0	0	0	0	0	0	0	1	1
Methylobacter	0	0	0	0	0	0	0	1	0	0	2	0	2	0	1	6	4
Deltaproteobacteria																	
Anaeromyxobacter	607	183	116	54	367	135	263	65	17	9	38	91	4	182	31	2,162	15
Smithella	4	71	1	4	5	0	1	0	8	5	2	0	0	3	12	116	11
Geobacter	5	0	0	0	2	0	0	0	0	0	0	1	0	3	0	11	4
Syntrophorhabdus	8	14	6	5	12	0	13	8	60	2	5	2	0	0	2	137	12
Epsilonproteobacteria																	
Sulfuricurvum	0	0	0	0	0	0	0	0	0	1,013	0	0	0	1	1	1,015	3

**Table A3:** Network global properties of alpine lake sediment samples microbial communities

Network indexes	Alpine lake sediment samples
Total nodes	167
Total links	256
R square of power‐law	0.937
Average degree (avgK)	3.066
Average clustering coefficient (avgCC)	0.089
Average path distance (GD)	6.584
Geodesic efficiency (*E*)	0.218
Harmonic geodesic distance (HD)	4.590
Maximal degree	16
Nodes with max degree	OTU_4
Centralization of degree (CD)	0.079
Maximal betweenness	3,027.276
Nodes with max betweenness	OTU_80
Centralization of betweenness (CB)	0.199
Density (*D*)	0.018
Reciprocity	1
Transitivity (Trans)	0.133
Connectedness (Con)	0.695
Efficiency	0.981
Hierarchy	0
Lubness	1
